# Xenomonitoring of Mosquitoes (Diptera: Culicidae) for the Presence of Filarioid Helminths in Eastern Austria

**DOI:** 10.1155/2018/9754695

**Published:** 2018-03-15

**Authors:** Sarah Susanne Übleis, Claudia Cuk, Michaela Nawratil, Julia Butter, Ellen Schoener, Adelheid G. Obwaller, Thomas Zechmeister, Georg G. Duscher, Franz Rubel, Karin Lebl, Carina Zittra, Hans-Peter Fuehrer

**Affiliations:** ^1^Institute of Parasitology, Department of Pathobiology, University of Veterinary Medicine Vienna, Veterinaerplatz 1, 1210 Vienna, Austria; ^2^Division of Science, Research and Development, Federal Ministry of Defence and Sports, Vienna, Austria; ^3^Biological Station Lake Neusiedl, Burgenland, Austria; ^4^Institute for Veterinary Public Health, Department for Farm Animals and Veterinary Public Health, University of Veterinary Medicine Vienna, Veterinaerplatz 1, 1210 Vienna, Austria

## Abstract

Information on mosquito-borne filarioid helminths in Austria is scarce, but recent discoveries of *Dirofilaria repens* indicate autochthonous distribution of this parasite in Eastern Austria. In the current xenomonitoring study, more than 48,000 mosquitoes were collected in Eastern Austria between 2013 and 2015, using different sampling techniques and storage conditions, and were analysed in pools with molecular tools for the presence of filarioid helminth DNA. Overall, DNA of *D. repens*, *Setaria tundra*, and two unknown filarioid helminths were documented in twenty mosquito pools within the mitochondrial *cox1* gene (barcode region). These results indicate that *S. tundra*, with roe deer as definite hosts, is common in Eastern Austria, with most occurrences in floodplain mosquitoes (e.g., *Aedes vexans*). Moreover, DNA of *D. repens* was found in an *Anopheles plumbeus* mosquito close to the Slovakian border, indicating that *D. repens* is endemic in low prevalence in Eastern Austria. This study shows that xenomonitoring is an adequate tool to analyse the presence of filarioid helminths, but results are influenced by mosquito sampling techniques, storage conditions, and molecular protocols.

## 1. Introduction

In Europe, filarioid helminths of veterinary and/or medical relevance have mainly been documented in Mediterranean regions, but increasingly these pathogens are being reported in temperate climate zones in Central and Northern Europe as well [1–3].

The most important filarioid helminths in Europe are *Dirofilaria immitis* and *D. repens*, causing canine pulmonary *(D. immitis)*, subcutaneous *(D. repens)*, and ocular (mainly *D. repens*) dirofilariosis [4]. Both, *D. immitis* and *D. repens*, are zoonotic parasites [4]. The first Central European discoveries of *D. immitis* were confirmed in Switzerland, in 1995 and 1998 [5, 6]. Since then, both parasites, *D. immitis* and *D. repens*, have been described in humans (accidental hosts), dogs (definite hosts), and mosquitoes (vectors) in many Central European countries. Both filarioid species have now been shown to be present in all countries neighbouring Austria except for Liechtenstein, namely, Switzerland, Italy, Slovenia, Hungary, Slovakia, the Czech Republic (*D. repens* only), and Germany [1, 3, 4, 7–11]. *D. repens* was documented in most Central European countries prior to *D. immitis*.

Mosquito-borne filarioid helminths of the genus *Setaria* mainly parasitize in the abdominal cavities of artiodactyls, hyracoids, and equines. Mosquitoes of the genus *Aedes* are thought to be the main vectors of these parasites (e.g., *Ae. vexans* for *S. labiatopapillosa*) [12, 13]. *Setaria tundra* is a parasite of roe deer documented in several European countries such as Austria, Switzerland, Germany, France, Italy, Hungary, Poland, Spain, and Denmark (summarized in Enemark et al. [14]). In Northern Europe, *S. tundra* can also be found in domestic reindeer, wild forest reindeer, and moose [15]. This species is associated with climate changes and causes severe outbreaks of periodontitis in semidomestic reindeer in Finland [16].

The aim of this study was to xenomonitor Eastern Austrian mosquitoes for the presence of DNA of filarioid helminths—with the main focus on *Dirofilaria repens* and *D. immitis* but also on *Setaria tundra* and other mosquito-borne filarioid helminths.

## 2. Materials and Methods

The present study combines the analysis for filarioid helminth DNA in mosquitoes sampled in two independent mosquito monitoring programs using two different storage conditions (dry and −80°C) conducted between 2013 and 2015.

### 2.1. Mosquito Sampling Method 1

In 2013 and 2014, adult female mosquitoes were trapped at three locations in Vienna using new standard miniature light traps (John W. Hook Company, Gainesville, Florida) baited with CO_2_. Collection was carried out on a daily basis for 24 hours from March to October. Mosquitoes were killed using the insecticide dichlorvos as soon as they entered the trap. Once a week, the traps were emptied, and Culicidae were dried and stored at room temperature until further processing [17].

### 2.2. Mosquito Sampling Method 2

Mosquitoes were monitored across three provinces of Eastern Austria (Burgenland, Lower Austria, and Vienna) at 35 permanent and 23 nonpermanent trapping sites. At permanent sampling sites, mosquitoes were monitored on a regular basis every second week for a 24-hour time period from April to October 2014-2015 using Biogents Sentinel Traps (Regensburg, Germany) equipped with carbon dioxide as attractant. Nonpermanent sampling sites were investigated at least once and up to six times during the summer months using Biogents Sentinel Traps (Regensburg, Germany) or exhausters. All mosquitoes were stored at −80°C until further processing [18].

Mosquitoes were identified morphologically using the identification key of Becker et al. [19] and pooled by species, collection site, and date, with a maximum number of 50 individuals per pool. To each pool, 400 *µ*l of DNA/RNA lysis buffer (Zymo Research Corp., USA) and two ceramic beads (Precellys Ceramic Beads, Peqlab Biotechnologie GmbH) were added, and the samples were homogenized in a TissueLyser II (Qiagen, Germany). Approximately 350 *µ*l of the homogenized pulp was loaded onto a QIAshredder (Qiagen, Germany). The filled QIAshredders were centrifuged for two minutes at 13,000 rpm to filter the samples (solid components remained on the column). In the next step, DNA was extracted using a ZR-Duet™ DNA/RNA MiniPrep kit (Zymo Research Corp., USA) according to the manufacturer's instructions.

DNA extracted from female mosquito pools was examined for the presence of genomic material of filarioid helminths using primers and PCR conditions published elsewhere [20]. The primers used target a 724 bp fragment of the mitochondrial cytochrome oxidase subunit I gene and are specific for various filarioid helminths (e.g., *Dirofilaria*, *Wuchereria*, *Brugia*, *Onchocerca*, *Setaria*, and *Acanthocheilonema*). PCR products were separated by electrophoresis in 2% agarose gels stained with Midori Green Advance DNA stain (Nippon Genetics Europe, Germany). Finally, purified PCR products were sequenced by a commercial company (LGC Genomics GmbH, Germany). Sequences thus obtained were compared for similarity to sequences available in GenBank® database (http://www.ncbi.nlm.nih.gov/BLAST).

## 3. Results and Discussion

**Figure 1:**
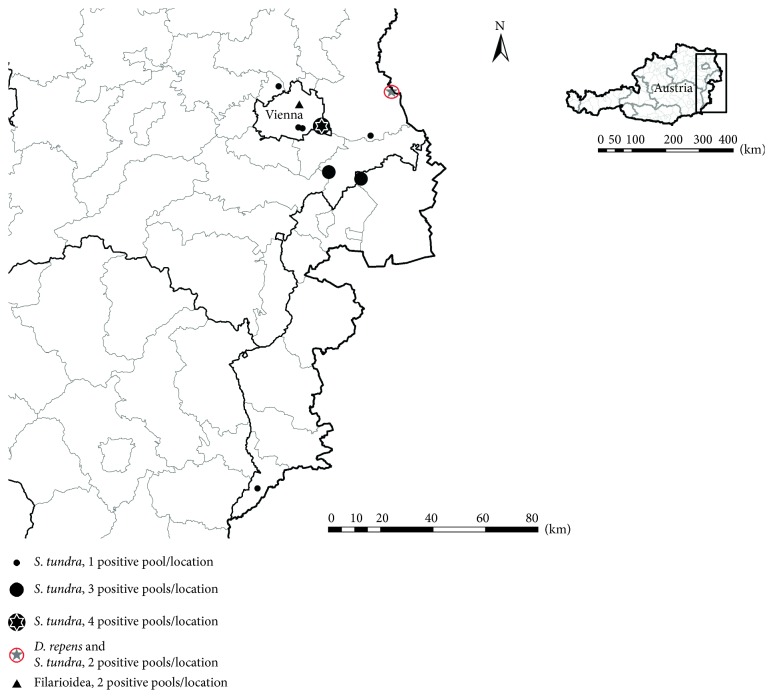
Geographic distribution of mosquito pools positive for filarioid helminths in Eastern Austria.

45,848 mosquitoes representing 25 mosquito species were analysed for the presence of filarioid DNA in this xenomonitoring survey (Table 1), resulting in the identification of DNA from *D. repens*, *S. tundra*, and two unknown filarioid helminths in 20 of the mosquito pools (Table 2; Figure 1).

DNA of *D. repens* was only found in 2015 in a single *Anopheles plumbeus* mosquito in Marchegg (Lower Austria) close to the Slovakian border. Although several mosquito species of different genera are proven as potential vectors of *D. repens* [21], DNA of this parasite has so far only been detected in other *Anopheles* species (*An. algeriensis* and *An. maculipennis* complex) in Austria [2].

To date, all *D. repens* positive mosquitoes have been collected in close proximity to the Slovakian (this study) and the Hungarian borders [2]. In both Slovakia and Hungary, *D. repens* is known to be endemic with a prevalence above 10% in dogs in the Bratislava area, close to the Austrian border [22, 23]. Previous metadata analysis has shown that most reported but also potential autochthonous findings in dogs were in Eastern Austria [10]. Furthermore, Duscher et al. [24] described the examination of *D. repens* positive dogs in the same districts where positive mosquitoes were documented (Gänserndorf and Neusiedl am See). This indicates that *D. repens* might be endemic with low prevalence in this area. Simon et al. [4] postulated that two preconditions are required for a successful establishment of *D. repens* and *D. immitis* in a novel area: (i) the presence of competent mosquito vectors, which is the case in Austria, and (ii) a certain number of positive dogs shedding microfilaria. The second precondition seems to limit the distribution of *D. repens* (but also *D. immitis*) because there are almost no stray dogs, and kennel holding is not common in Austria.

*Dirofilaria immitis* was not identified in the present large-scale survey, confirming previous results that this parasite has not yet established itself in Eastern Austria [10]. This pathogen has however been confirmed in dogs [22, 23, 25] and in mosquitoes [26, 27] in Slovakia and Hungary in the vicinity of our study area.

The most commonly found filarioid helminth within the present study area in Eastern Austria was *S. tundra*, with most occurrences of *S. tundra* DNA in mosquitoes of the genus *Aedes*, especially *Ae. vexans*. Similarly, prevalences of up to 12.3% have been reported in roe deer in Central Europe (e.g., northern Bavaria [28]). This parasite has also been recorded in *Ae. vexans* in studies in Germany and Hungary [1, 27, 29, 30], suggesting that *S. tundra* is a common parasite of roe deer in Eastern Austria.

The discovery of DNA of unknown filarioid helminths in ornithophilic *Culex* mosquitoes (*Cx. modestus* and *Cx. pipiens* complex) is not surprising because several avian filarioid helminths (with low pathogenicity for bird hosts) are present in Central Europe [29].

## 4. Conclusions

This xenomonitoring survey confirms the presence of DNA of certain filarioid helminths in mosquitoes in Eastern Austria and indicates possible vector competence of select mosquito species. However, comparison of the two sampling techniques and storage schemes used here suggests that storage of dried mosquitoes at room temperature increases the number of false negative pools because of a decrease of DNA quality, a circumstance that has also been noticed during analyses of mosquito DNA itself [31]. Moreover, different techniques for mosquito sampling (certain mosquito species are attracted by certain traps) and the use of different PCR protocols also influence the outcome of xenomonitoring studies [32]. Nevertheless, xenomonitoring is an effective tool to examine if certain pathogens are present in an area (e.g., [27]). It can be concluded that *D. repens*, *S. tundra*, and unknown filarioid helminths (most probably avian parasites) are present in Eastern Austria. Further studies are needed to monitor in more detail the situation of *D. repens* and *D. immitis* in Austria and neighbouring countries.

## Figures and Tables

**Table 1:** Number of mosquitoes and species collected in Eastern Austria included within this study.

Mosquito species	2013 HC	2014 HC	2014 Biodiversa	2015 Biodiversa	Total
*Aedes cinereus*/*geminus*	6	6	299	33	344
*Aedes vexans*	1718	1847	4417	1179	9161
*Anopheles algeriensis*	4	—	—	3	7
*Anopheles claviger*	—	—	—	13	13
*Anopheles hyrcanus*	145	63	—	241	449
*Anopheles maculipennis* complex	14	2	13	41	70
*Anopheles plumbeus*	9	26	150	196	381
*Coquillettidia richiardii*	2169	4333	1287	8034	15823
*Culex martinii*	—	—	66	996	1062
*Culex modestus*	31	8	—	61	100
*Culex pipiens* complex/*Cx. torrentium*	2707	2090	2118	7124	14039
*Culex territans*	—	8	—	—	8
*Culiseta annulata*	4	7	—	33	44
*Ochlerotatus cantans*	—	—	1	1	2
*Ochlerotatus caspius*	—	51	17	4	72
*Ochlerotatus cataphyla*	—	—	7	5	12
*Ochlerotatus communis*	—	—	22	—	22
*Ochlerotatus flavescens*	—	—	1	—	1
*Ochlerotatus geniculatus*	4	41	20	6	71
*Ochlerotatus intrudens*	—	—	24	—	24
*Ochlerotatus japonicus japonicus*	—	—	—	6	6
*Ochlerotatus leucomelas*	—	—	—	1	1
*Ochlerotatus rusticus*	—	—	4	—	4
*Ochlerotatus sticticus*	559	202	1113	499	2373
*Uranotaenia unguiculata*	—	—	—	10	10
*Aedes/Ochlerotatus* sp.	^a^	^a^	735	217	952
*Anopheles* sp.	^a^	^a^	22	387	409
*Culex* sp.	^a^	^a^	131	257	388
Total	**7370**	**8684**	**10447**	**19347**	**45848**

HC, Hook Company Traps; Biodiversa, combination of various traps including BG-Sentinel Traps, HC, and aspirators; ^a^mosquitoes specified to genus level were not included.

**Table 2:** Filarioid helminths in mosquitoes in Eastern Austria: Vienna, Lower Austria (LA), and Burgenland (B).

Filarioid species	Mosquito species	Collection site	Sampling method	Collection date	Pool size	GenBank entry	Maximum % identity to GenBank entries^b^
*Dirofilaria repens*	*Anopheles plumbeus*	Marchegg (LA)	BG-Sentinel	August 2015	1	MF695085	100%
*Setaria tundra*	*Aedes cinereus*/*geminus*	Lobau (Vienna)	HC^a^	July 2013	1	MF695086	100%
*Setaria tundra*	*Aedes vexans*	Marchegg (LA)	BG-Sentinel	July 2014	25	MF695087	>99%
*Setaria tundra*	*Aedes* sp.	Lobau (Vienna)	HC^a^	August 2014	9	MF695088	>99%
*Setaria tundra*	*Aedes vexans*	Lobau (Vienna)	HC^a^	August 2014	42	MF695089	100%
*Setaria tundra*	*Coquillettidia richiardii*	Lobau (Vienna)	HC^a^	August 2014	50	MF695090	>99%
*Setaria tundra*	*Aedes vexans*	Vienna	Aspirator	August 2014	1	MF695091	100%
*Setaria tundra*	*Aedes vexans*	Marchegg (LA)	BG-Sentinel	August 2014	3	nd^c^	100%
*Setaria tundra*	*Aedes cinereus*/*geminus*	Eckartsau (LA)	BG-Sentinel	June 2015	1	MF695096	>99%
*Setaria tundra*	*Aedes* sp.	Klosterneuburg (LA)	BG-Sentinel	June 2015	25	nd^c^	100%
*Setaria tundra*	*Culex pipiens* complex	Bruckneudorf (B)	BG-Sentinel	July 2015	50	nd^c^	100%
*Setaria tundra*	*Aedes* sp.	Götzendorf (LA)	BG-Sentinel	July 2015	6	MF695092	99%
*Setaria tundra*	*Aedes vexans*	Götzendorf (LA)	BG-Sentinel	July 2015	50	MF695093	100%
*Setaria tundra*	*Aedes vexans*	Götzendorf (LA)	BG-Sentinel	July 2015	50	nd^c^	100%
*Setaria tundra*	*Aedes vexans*	Jennersdorf (B)	BG-Sentinel	July 2015	1	MF695094	100%
*Setaria tundra*	*Aedes* sp.	Bruckneudorf (B)	BG-Sentinel	July 2015	4	nd^c^	100%
*Setaria tundra*	*Aedes vexans*	Bruckneudorf (B)	BG-Sentinel	July 2015	26	MF695095	100%
*Setaria tundra*	*Culex pipiens* complex	Vienna	BG-Sentinel	August 2015	50	nd^c^	100%
Filarioidea	*Culex modestus*	Vienna	HC^a^	June 2014	1	nd^c^	93%
Filarioidea	*Culex pipiens* complex	Vienna	HC^a^	September 2014	1	nd^c^	95%

^a^Hook Company CO_2_ baited mosquito traps; ^b^analysis of maximum identity to GenBank Entries was performed on August 4, 2017; ^c^sequences were not uploaded to GenBank (e.g., short sequences or poor sequence quality).

## References

[1] Kronefeld M., Kampen H., Sassnau R., Werner D. (2014). Molecular detection of *Dirofilaria immitis*, *Dirofilaria repens* and *Setaria tundra* in mosquitoes from Germany. *Parasites & Vectors*.

[2] Silbermayr K., Eigner B., Joachim A. (2014). Autochthonous *Dirofilaria repens* in Austria. *Parasites & Vectors*.

[3] Matějů J., Chanová M., Modrý D. (2016). *Dirofilaria repens*: emergence of autochthonous human infections in the Czech Republic (case reports). *BMC Infectious Diseases*.

[4] Simón F., Siles-Lucas M., Morchón R. (2012). Human and animal dirofilariasis: the emergence of a zoonotic mosaic. *Clinical Microbiology Reviews*.

[5] Deplazes P., Guscetti F., Wunderlin E., Bucklar H., Skaggs J., Wolff K. (1995). Endoparasite infection in stray and abandoned dogs in southern Switzerland. *Schweizer Archiv für Tierheilkunde*.

[6] Bucklar H., Scheu U., Mossi R., Deplazes P. (1998). Is dirofilariasis in dogs spreading in south Switzerland?. *Schweizer Archiv für Tierheilkunde*.

[7] Genchi C., Kramer L. H., Rivasi F. (2011). Dirofilarial infections in Europe. *Vector-Borne and Zoonotic Diseases*.

[8] Bocková E., Rudolf I., Kočišová A. (2013). *Dirofilaria repens* microfilariae in *Aedes vexans* mosquitoes in Slovakia. *Parasitology Research*.

[9] Czajka C., Becker N., Jöst H. (2014). Stable transmission of *Dirofilaria repens* nematodes, northern Germany. *Emerging Infectious Diseases*.

[10] Fuehrer H. P., Auer H., Leschnik M., Silbermayr K., Duscher G., Joachim A. (2016). *Dirofilaria* in humans, dogs, and vectors in Austria (1978-2014)-from imported pathogens to the endemicity of *Dirofilaria repens*. *PLoS Neglected Tropical Diseases*.

[11] Miterpáková M., Antolová D., Ondriska F., Gál V. (2017). Human *Dirofilaria repens* infections diagnosed in Slovakia in the last 10 years (2007-2017). *Wiener Klinische Wochenschrift*.

[12] Laaksonen S., Solismaa M., Kortet R., Kuusela J., Oksanen A. (2009). Vectors and transmission dynamics for *Setaria tundra* (Filarioidea; Onchocercidae), a parasite of reindeer in Finland. *Parasites & Vectors*.

[13] Ionică A. M., Zittra C., Wimmer V. (2017). Mosquitoes in the Danube Delta: searching for vectors of filarioid helminths and avian malaria. *Parasites & Vectors*.

[14] Enemark H. L., Oksanen A., Chriél M., le Fèvre Harslund J., Woolsey I. D., Al-Sabi M. N. (2017). Detection and molecular characterization of the mosquito-borne filarial nematode *Setaria tundra* in Danish roe deer (*Capreolus capreolus*). *International Journal for Parasitology: Parasites and Wildlife*.

[15] Laaksonen S., Solismaa M., Orro T. (2009). *Setaria tundra* microfilariae in reindeer and other cervids in Finland. *Parasitology Research*.

[16] Laaksonen S., Kuusela J., Nikander S., Nylund M., Oksanen A. (2007). Outbreak of parasitic peritonitis in reindeer in Finland. *Veterinary Record*.

[17] Lebl K., Zittra C., Silbermayr K. (2015). Mosquitoes (Diptera: Culicidae) and their relevance as disease vectors in the city of Vienna, Austria. *Parasitology Research*.

[18] Zittra C., Vitecek S., Obwaller A. G. (2017). Landscape structure affects distribution of potential disease vectors (Diptera: Culicidae). *Parasites & Vectors*.

[19] Becker N., Petric D., Zgomba M. (2010). *Mosquitoes and Their Control*.

[20] Hodžić A., Alić A., Fuehrer H. P., Harl J., Wille-Piazzai W., Duscher G. G. (2015). A molecular survey of vector-borne pathogens in red foxes (*Vulpes vulpes*) from Bosnia and Herzegovina. *Parasites & Vectors*.

[21] Silaghi C., Beck R., Capelli G., Montarsi F., Mathis A. (2017). Development of *Dirofilaria immitis* and *Dirofilaria repens* in *Aedes japonicus* and *Aedes geniculatus*. *Parasites & Vectors*.

[22] Miterpáková M., Iglódyová A., Čabanová V., Stloukal E., Miklisová D. (2016). Canine dirofilariosis endemic in Central Europe—10 years of epidemiological study in Slovakia. *Parasitology Research*.

[23] Trájer A., Rengei A., Farkas-Iványi K., Bede-Fazekas A. (2016). Impacts of urbanisation level and distance from potential natural mosquito breeding habitats on the abundance of canine dirofilariosis. *Acta Veterinaria Hungarica*.

[24] Duscher G., Feiler A., Wille-Piazzai W. (2009). Detection of *Dirofilaria* in Austrian dogs. *Berliner und Münchener tierärztliche Wochenschrift*.

[25] Bacsadi A., Papp A., Szeredi L. (2016). Retrospective study on the distribution of *Dirofilaria immitis* in dogs in Hungary. *Veterinary Parasitology*.

[26] Bocková E., Iglódyová A., Kočišová A. (2015). Potential mosquito (Diptera:Culicidae) vector of *Dirofilaria repens* and *Dirofilaria immitis* in urban areas of Eastern Slovakia. *Parasitology Research*.

[27] Zittra C., Kocziha Z., Pinnyei S. (2015). Screening blood-fed mosquitoes for the diagnosis of filarioid helminths and avian malaria. *Parasites & Vectors*.

[28] Büttner K. (1978). Untersuchungen zur Parasitierung des Rehwildes bei steigendem Jagddruck. *Zeitschrift für Jagdwissenschaft*.

[29] Czajka C., Becker N., Poppert S., Jöst H., Schmidt-Chanasit J., Krüger A. (2012). Molecular detection of *Setaria tundra* (Nematoda: Filarioidea) and an unidentified filarial species in mosquitoes in Germany. *Parasites & Vectors*.

[30] Kemenesi G., Kurucz K., Kepner A. (2015). Circulation of *Dirofilaria repens, Setaria tundra*, and Onchocercidae species in Hungary during the period 2011-2013. *Veterinary Parasitology*.

[31] Werblow A., Flechl E., Klimpel S. (2016). Direct PCR of indigenous and invasive mosquito species: a time- and cost-effective technique of mosquito barcoding. *Medical and Veterinary Entomology*.

[32] Masny A., Sałamatin R., Rozej-Bielicka W., Golab E. (2016). Is molecular xenomonitoring of mosquitoes for *Dirofilaria repens* suitable for dirofilariosis surveillance in endemic regions?. *Parasitology Research*.

